# Accessing Plasmonic Hotspots Using Nanoparticle-on-Foil Constructs

**DOI:** 10.1021/acsphotonics.1c01048

**Published:** 2021-08-23

**Authors:** Rohit Chikkaraddy, Jeremy J Baumberg

**Affiliations:** † NanoPhotonics Centre, Cavendish Laboratory, Department of Physics, JJ Thompson Avenue, University of Cambridge, Cambridge CB3 0HE, United Kingdom

**Keywords:** plasmonic cavity, polaritons, atomic monolayer, thin films, SERS, antenna

## Abstract

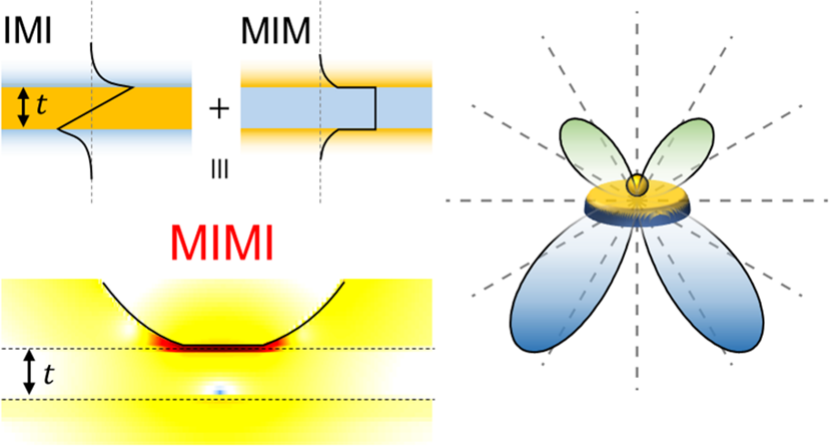

Metal–insulator–metal (MIM) nanogaps in the canonical nanoparticle-on-mirror geometry (NPoM) provide deep-subwavelength confinement of light with mode volumes smaller than *V*/*V*_λ_ < 10^–6^. However, access to these hotspots is limited by the impendence mismatch between the high in-plane *k*_∥_ of trapped light and free-space plane-waves, making the in- and out-coupling of light difficult. Here, by constructing a nanoparticle-on-foil (NPoF) system with thin metal films, we show the mixing of insulator–metal–insulator (IMI) modes and MIM gap modes results in MIMI modes. This mixing provides multichannel access to the plasmonic nanocavity through light incident from both sides of the metal film. The red-tuning and near-field strength of MIMI modes for thinner foils is measured experimentally with white-light scattering and surface-enhanced Raman scattering from individual NPoFs. We discuss further the utility of NPoF systems, since the geometry allows tightly confined light to be accessed simply through different ports.

Despite the recent realization that visible light can be trapped using metals to the nanometer scale and below (mode volumes *V* < 100 nm^3^), efficient access to these “hotspots” gives many challenges.^1,2^ Essentially, the requirement is for impedance matching through an antenna^3−5^ so that coupling is then much better than the simple overlap integral *V*/λ^3^ < 10^–6^. While surface plasmon polaritons (which confine on the 10–100 nm length scale) can be accessed using grating or prism couplers, this is not efficient with tighter confinement,^6−9^ because the in-plane *k*_∥_ required is so large.

At the heart of metal-confining light architectures are layered structures with subsequent lateral patterning. Typically, these layers are based around two variants: (i) insulator–metal–insulator (IMI) sheets or (ii) metal–insulator–metal (MIM) gaps. In IMI systems, SPPs on either side of a metal film strongly hybridize when the metal thickness (*t*) is smaller than its skin depth (<20 nm), resulting in large in-plane wave-vector (*k*_∥_) modes confining EM fields close to the metal sheet. Recently, such modes were identified in two-dimensional graphene as well as metallic transition metal dichalcogenide (TMD) monolayers above their plasma wavelength (4–12 μm) and were utilized to achieve high confinement as well as molecular sensing.^10−13^ In MIM gaps, the SPPs on either side of the dielectric medium similarly hybridize to form symmetric gap modes with large *k*_∥_. Such confined light between two metal surfaces in a narrow gap has been extensively utilized for sensing,^14,15^ Purcell-enhanced emission from single emitters,^16,17^ strong coupling,^18^ surface-enhanced Raman scattering (SERS),^19^ and in metamaterials.^20^

Crucial to obtain tight confinement in both cases is to achieve reliable sub-nm film thicknesses (*t*) or gaps (*d*) capable of producing large *n*_eff_ (>100), which corresponds to effective plasmon wavelengths of <10 nm. While the thinnest metal sheets have reached *t* ≈ 3 nm,^21−23^ it has been possible using TMD or molecular spacers to routinely achieve *d* < 1 nm gaps.^24^ Convenient lateral confinement at the 10 nm scale can then be produced using nanoparticle (NP) facets created using colloidal assembly (in MIMs between NPs in dimers^25−27^), though top-down fabrication also aims to access the same domain.^28,29^ A particularly reliable, scalable, and robust construct for MIM geometries uses nanoparticle-on-mirror (NPoM) systems,^30,31^ where a colloidally synthesized (usually single-crystal gold) nanoparticle is placed on top of an atomically flat >100-nm-thick mirror, which is predeposited with a self-assembled monolayer of molecules. This results in an MIM geometry with gap size set by the thickness of the molecular layer (*d* < 2 nm), identical across large areas (>100 cm^2^).^32^ However, access to these hotspots is confounded by the thick nontransparent Au mirror, restricting its utilization.

In this work, we present a nanoparticle-on-foil geometry (NPoF) with a finite thickness Au substrate providing both front and back access to the confined light in the nanogap. It arises from the mixed coupling of both IMI and MIM modes across the thin Au foil, resulting in new metal–insulator–metal–insulator (MIMI) modes. By tuning the film thickness, we modify the effective index of these MIMI gaps and control the far-field scattering and near-field SERS.

In previous work on related NPoF geometries with film thickness >20 nm or gaps >5 nm, the nanocavity modes were not understood to depend on film thickness, even though the gap is accessible with the excitation of surface plasmon polaritons (SPPs) on the metal film through prism coupling.^33−36^ By making even thinner metal films, we might expect the effective image dipole coupling would fade out and coupled plasmon cavity modes would damp out. However, surprisingly, we find in NPoF systems here with *t* < 10 nm and *d* < 2 nm that the coupled SPPs on either side of the foil mix with MIM modes trapped in the gap, forming MIMI modes.

## Coupling of IMI and MIM Modes

**Figure 1:**
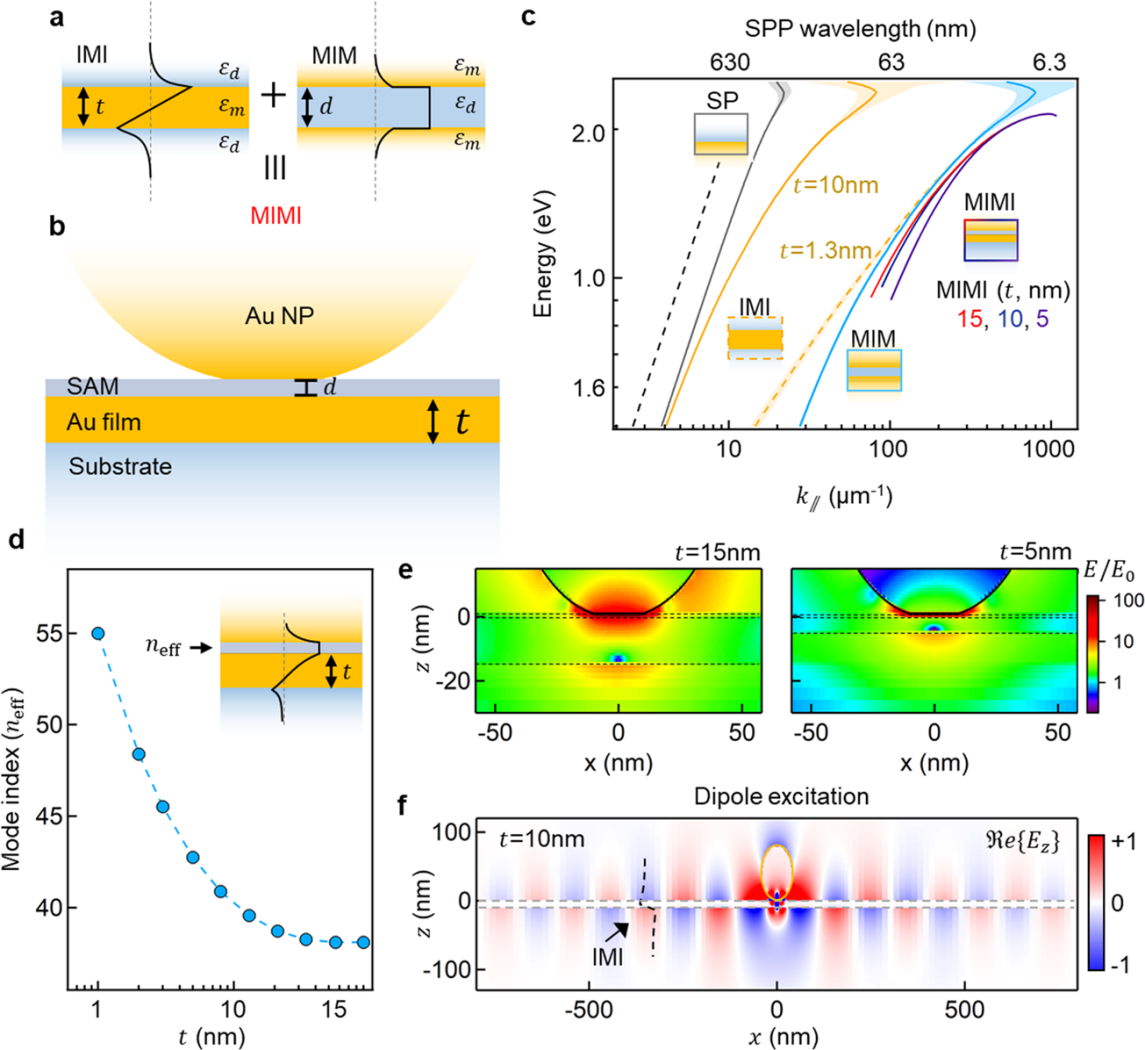
Coupling of IMI + MIM = MIMI modes in a nanoparticle-on-foil cavity. (a) Tight mode confinement in IMI and MIM geometries. Black curves show ***H*_*y*_** in thin film (*t*) and narrow gap (*d*) limits. (b) Schematic nanoparticle-on-foil (NPoF) geometry here combines MIM and IMI modes into a single architecture of hybridized MIMI modes. (c) Dispersion of MIMI (red-purple) modes for different *t* with *d* = 1 nm compared to IMI (yellow, *t* = 10 nm solid, *t* = 1.3 nm dashed) and MIM (blue, *d* = 1.3 nm) modes; surface plasmon on thick Au (gray) and free-space photon (dashed gray). (d) Effective propagation index of MIMI modes for increasing *t* at λ = 633 nm. (e) ***E*_*z*_** near-fields from full-wave simulations of NPoF with plane-wave excitation for *t* = 15 nm and *t* = 5 nm film thickness at the nanocavity resonance. The field null in the metal film just above the substrate and the leakage of nanocavity fields into the substrate are clearly seen. (f) ***E*_*z*_** near-fields from full-wave simulations of NPoF geometry with a dipole source exciting the NPoF gap center for *t* = 10 nm at λ = 633 nm, highlighting IMI modes traveling away from the AuNP.

Before discussing the coupling of MIM and IMI modes (Figure 1a,b), we need to compare the key differences between MIM and IMI plasmon mode dispersions. The explicit IMI dispersion in the thin film limit is given by^37,38^
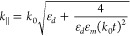
1where . Here, *ε*_*m*_ (ω) and *ε*_*d*_ are the dielectric functions of the metal and the dielectric, respectively. The values obtained from the above equation match well with the full solution (SI). The dispersion of an MIM mode in the narrow gap limit is given by refs (39−41) (see Supporting Information A)
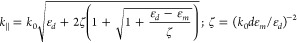
2When *d* or *t* is large (>50 nm), these dispersions are not significantly different from that of an SPP on a single Au surface. However, when *d*,*t* < 5 nm, the dispersions become flat with extremely small plasmon wavelengths (<10 nm). For *d* = *t*, IMI and MIM dispersions converge to the same *n*_eff_ = *k*_∥_/*k*_0_ at large *k* (Figure 1c).

In the NPoM geometry, the facet width *w* of the AuNP on top defines the lateral discretization of the MIM modes. Essentially, this forms a type of Fabry–Perot resonator with solutions *k*_∥_*w* = *nπ* for 1D (and similar Bessel function solutions in 2D)^42,43^ formed by the reflections on either end due to the large mismatch between *k*_∥_ in the MIM gap and *k*_0_ in free space.^42,44^ However, these resonant hotspots in the gap under the NP are relatively inaccessible and typically emit into high angles for small *d*, thus compromising efficient coupling.

This can be modified through hybridizing MIM and IMI modes by reducing the metal thickness to *t* < 20 nm (Figure 1c). The IMI fields at the dielectric substrate/Au interface then interact with the MIM gap fields trapped in the AuNP-gap-Au film, resulting in MIMI modes with *n*_eff_ > 50 for *t* < 5 nm (Figure 1d). Full-wave simulations for NPoFs with a dipole excitation source in the middle of the MIM gap confirm the launching of IMI modes away from the NPoF (Figure 1e,f). The effective wavelength of IMI modes is tuned as the film thickness reduces (Supporting Information, Figure S1). It is important to note that without the AuNP, the magnitude of IMI fields launched is 8000× lower, and this is because the radiation from the dipole in the MIM gap is Purcell-enhanced.

As the gap reduces, the intense local field inside the MIM gap leaks further underneath the foil, producing an accessible hotspot in the dielectric substrate (Figure 1e). As we show below, this can be made available for a variety of coupling schemes.

## Far-Field Scattering from MIMI Gaps

**Figure 2:**
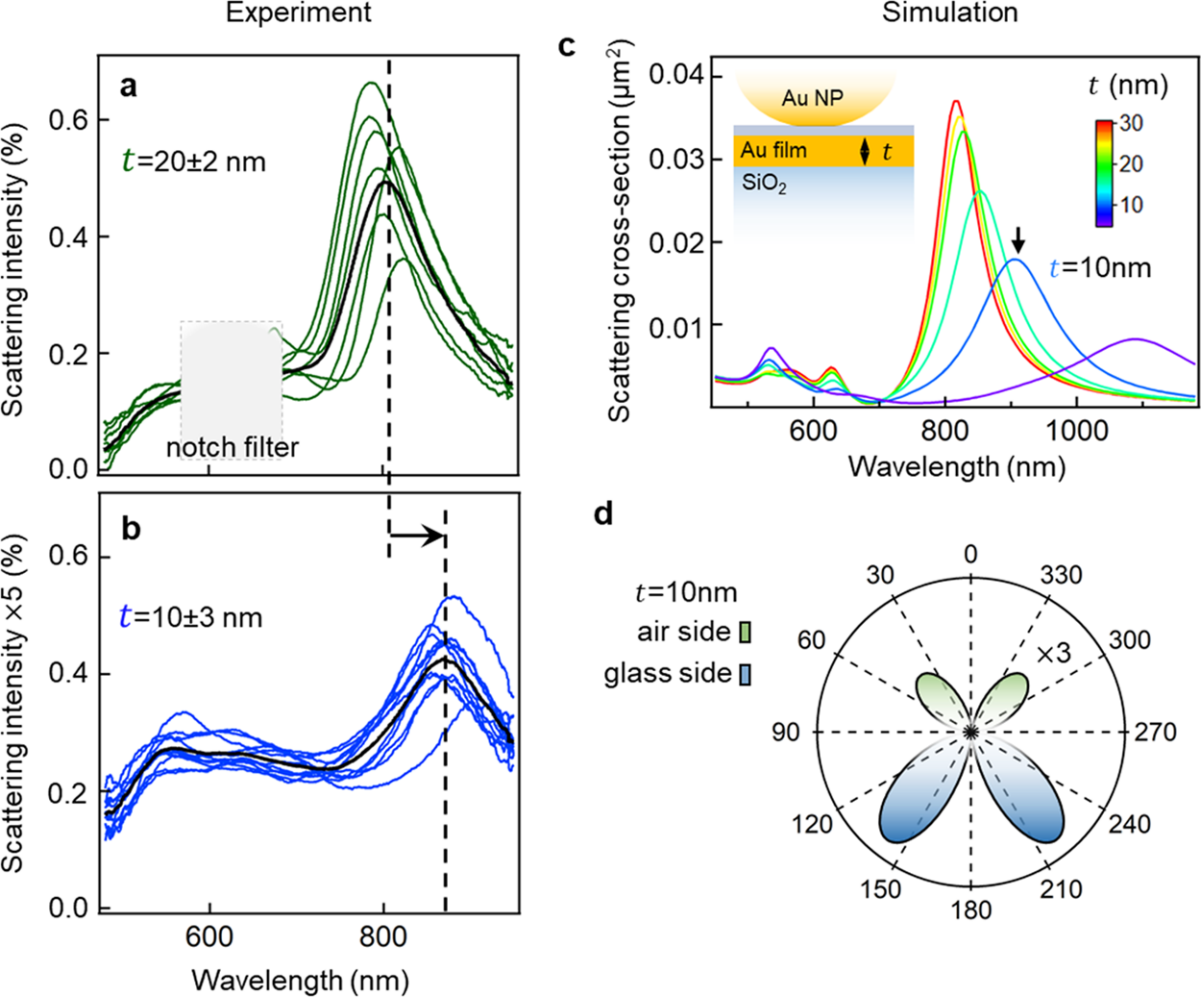
Far-field scattering from NPoFs. (a,b) Dark-field scattering spectra obtained from 10 different individual NPoF cavities for (a) *t* = 20 nm and (b) *t* = 10 nm. The black curve represents the average spectra. The red-shift in the scattering resonance is indicated by the horizontal arrow; notch laser filter for Raman shown in gray. (c) Simulated scattering spectra for NPoF cavities *vs* decreasing foil thickness *t* from 30 to 5 nm (black arrow marks *t* = 10 nm). (d) Simulated far-field radiated intensity on a polar plot for NPoF cavity, showing both air-side (scaled by ×3) and glass-side emission.

To characterize optical coupling into these localized MIMI modes, we fabricate NPoF samples with different thicknesses of the Au foil on a glass substrate. The AuNPs of diameter 2*R* = 80 nm are deposited on top of a compact self-assembled monolayer of BPT molecules (*d* = 1.3 nm), and the dark-field scattering spectra from individual Au NPoFs are collected using a custom-built white-light microscope. For the *t* = 20 ± 2 nm foil, the dominant scattering resonance is observed at 805 ± 10 nm, but when the foil thickness is reduced to 10 nm, the resonance shifts to 890 nm (Figure 2a,b). Intuitively, this red-shift is surprising, because a thinner confining metal foil under the NPoM would be expected to decrease the optical confinement, thus leading to blue-shifts. The origin of this is the asymmetric MIMI mode shape (Figure 1d, inset), which, in fact, increases the confinement due to the field null inside the metal foil. Intuitively, this can be also understood from the mixing between IMI and MIM modes that produces a lower energy coupled mode, thus shifting it to a higher wavenumber (Figure 1c) that results in a faster exponential decay into the metal, thus increasing confinement.^30^ The red-shift is >10 standard deviations outside the spectral shifts arising from AuNP size and shape variations, while the observed scattering intensity is 10× lower for *t* = 10 nm foils compared to *t* = 20 nm at resonance.

Two key features are observed in the experimental data shown in Figure 2a,b. First, there is the shift in the plasmon resonance with foil thickness, and second, there is the change in the mode intensity. These experimental observations are corroborated with 3D full-wave simulations (see Methods) using the experimental geometry. As *t* is tuned from 30 to 10 nm, the NPoF scattering resonance shifts gradually from 815 to 905 nm (Figure 2c), matching the experiments. A further reduction in *t* shifts the resonance beyond λ = 1 μm. This red-shift is consistent with the estimated change in *n*_eff_ for MIMI modes and can be understood from the MIM to MIMI dispersions (Figure 1d), which for fixed *k*_∥_ = π/*w* yield red-shifted resonances. The ±10 nm discrepancy observed between simulation and experiment is associated with the exact nanoparticle shape and gap morphology. A poorer agreement is found for scattering intensities, with simulations showing only a 2-fold decrease on halving *t*, which contrasts with the 10-fold reduction observed in experiments. To understand this, we calculate the far-field radiation pattern of light scattering from an NPoF to both air- and glass-sides (Figure 2d). Unsurprisingly, the light now leaks into the glass-side beyond the total-internal reflection angle (TIR = 45°) when *t* is below the skin depth of Au (10 nm). For *t* = 10 nm, the light scattered into the air is 4.5× times smaller than the light leaking to the glass-side, which thus accounts for the observed 10-fold decrease in experiments (scattered light is collected only on the air-side with NA = 0.9). We also confirm this experimentally using a modified dual channel microscope with white laser illuminating wide-field areas of the sample at high incident angles and the scattered light collected separately on air- and glass-sides (Supporting Information, Figure S2). Note that the fraction of light leaking to the glass-side can reach >90% for *t* < 5 nm.

## Near-Field and SERS

To characterize the near-field strength of these modes, surface-enhanced Raman scattering (SERS) signals are obtained from the self-assembled monolayer of biphenyl-4-thiol (BPT) molecules assembled between the AuNP and Au film. Individual NPoFs are illuminated from the air-side with a 633 nm laser, and backscattered Stokes light is measured.

**Figure 3:**
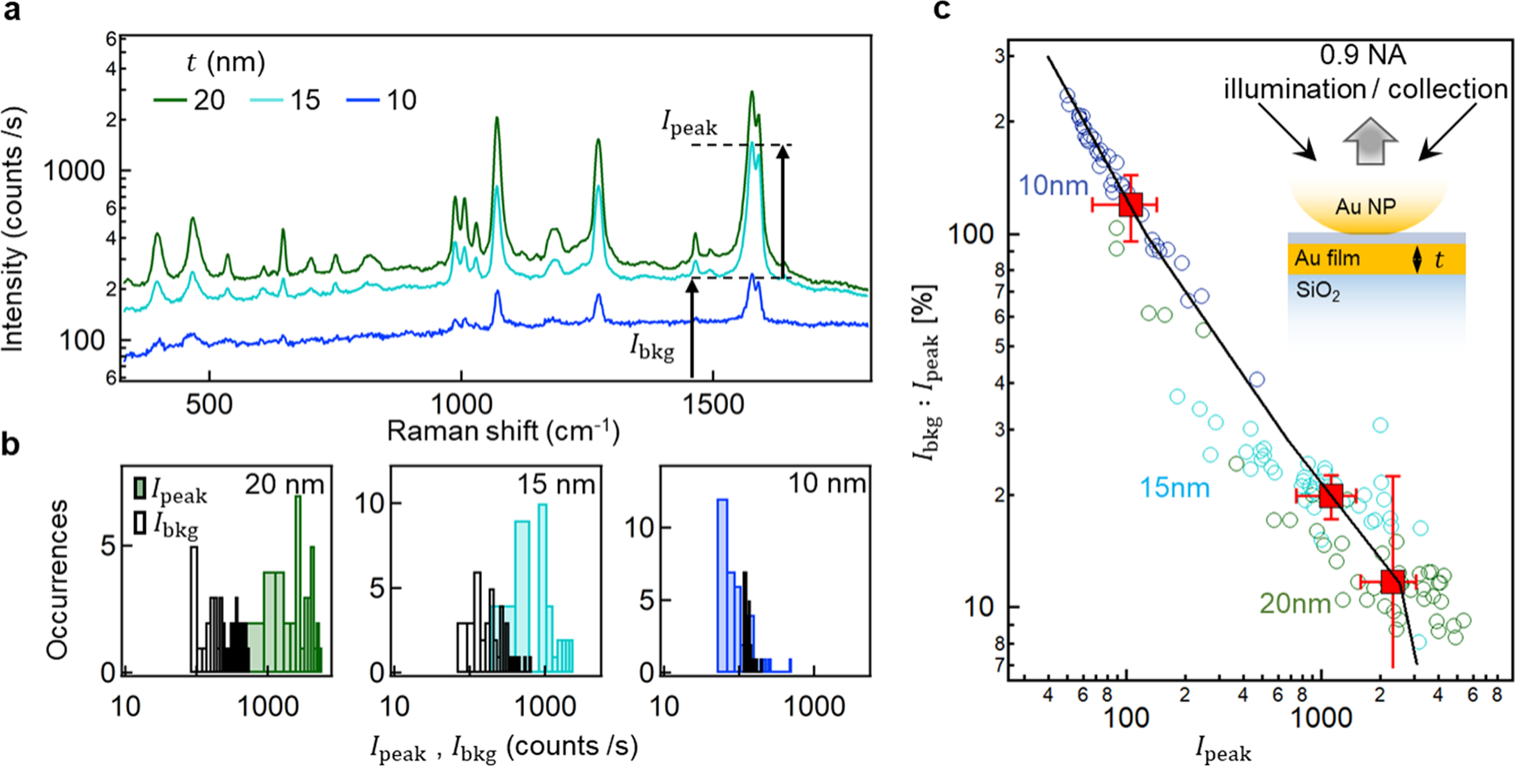
SERS enhancements in NPoFs. (a) Average SERS spectra obtained from 30 individual NPoFs for 3 different film thicknesses. The SERS intensity is plotted on a log-scale. (b) Histogram of extracted SERS 1585 cm^–1^ peak and background intensities for 30 individual NPoFs. (c) Variation in the ratio of SERS background to SERS peak intensities. Data from different *t* are color coded to (b), and average values for each *t* are shown as red points along with their standard error. Black line shows prediction from full-wave simulations.

The average SERS spectra from 30 different NPoFs for 3 different foil *t* are shown in Figure 3a (note SERS intensity is plotted on a log-scale). The observed SERS signals contain sharp narrow peaks of widths <15 cm^–1^ corresponding to different Raman vibrational modes of BPT molecules and a broad inelastic background from the electronic Raman scattering (ERS) of conduction band electrons in both AuNP and Au foil. Intensities from these two processes are quantified by Gaussian fits to the BPT vibrations to extract their peak height (*I*_peak_) as well as the SERS background (*I*_bkg_). Histograms of *I*_peak_ and *I*_bkg_ from 30 NPoFs reveal a key feature that as *t* decreases from 20 to 10 nm; the overall SERS intensity dramatically decreases, and *I*_peak_ drops by >20×. This cannot be attributed to the shift in plasmon resonance position, as on- *vs* off-resonance excitation changes the intensity only by a factor of 5.

For practical sensing, the important quantity is the ratio of peak SERS to background as well as how this ratio responds to the tuning of fields inside and outside the metal. To quantify these variations, the ratio *I*_bkg_:*I*_peak_ is plotted *vs I*_peak_ (Figure 3c). While for *t* = 20 nm there is only 10% contribution from the metal electronic SERS compared to the molecular SERS, this reaches *I*_bkg_ ≥ *I*_peak_ for *t* = 10 nm, arising from the greater penetration of light into the Au foil, as shown below.

Near-field enhancement factors are extracted from wavelength-dependent full-wave simulations (see Methods). The near-field enhancement (*E*/*E*_0_) extracted from the center of the NPoF with a BPT monolayer decreases for smaller *t* and follows closely the scattering cross section (Figure 2c). The simulations for normal illumination show similar trends (Supporting Information, Figure S3). The change in *I*_SERS_ ∝ (*E*_*in*_^2^/*E*_0_^2^) × (*E*_*out*_^2^/*E*_0_^2^) in the in-/out-going wavelength range of 600–750 nm multiplied by the fraction directed to the air-side accounts for the 25-fold decrease in the experimental SERS intensity as *t* is decreased from 20 to 10 nm (Figure 3c, Supporting Information, Figure S4). It is also apparent that the NPoF cavity resonance is slightly more damped for thin foils but still clearly present.

**Figure 4:**
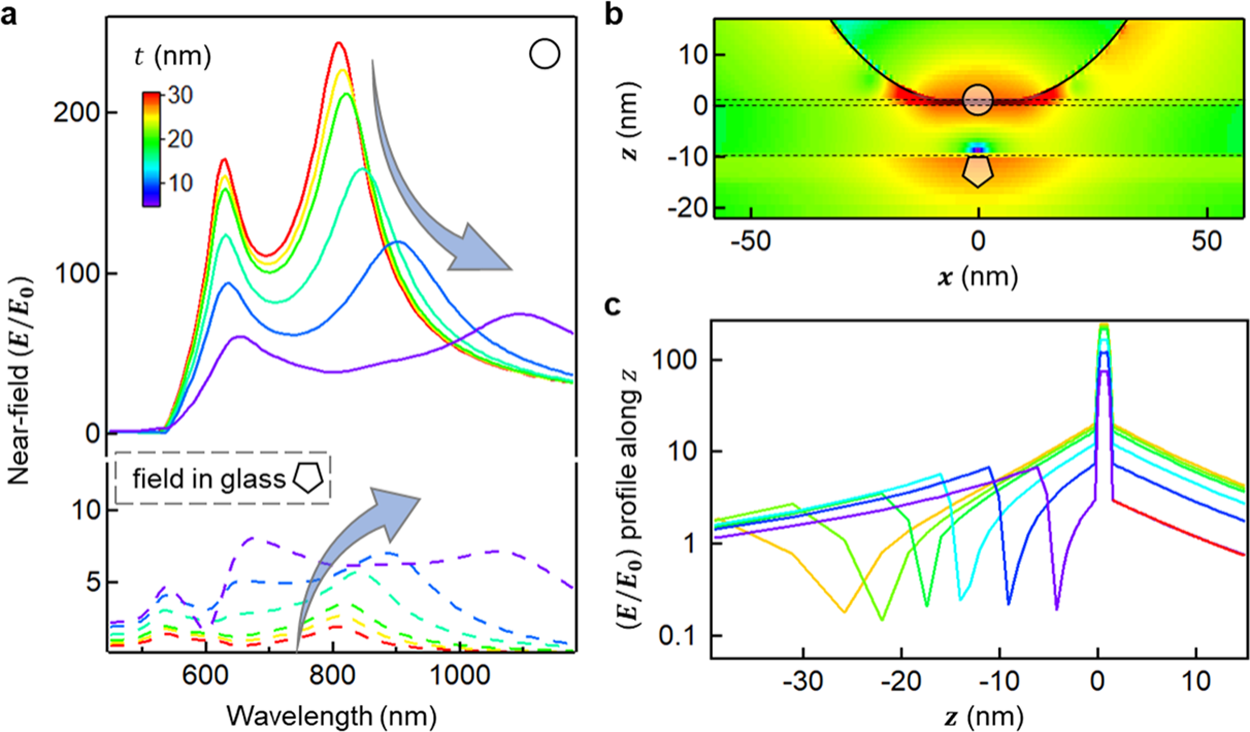
Near-field enhancements in NPoFs. (a) Simulated wavelength-dependent near-field intensities extracted from the center of the BPT-gap layer and fields 2 nm below the Au foil (indicated by circle and pentagon symbols in (b)) for different film thicknesses (NPoF is illuminated with plane-wave with polarization perpendicular to the film surface at a glancing angle of 90°). (b) Near-field enhancement map for *t* = 10 nm at the nanocavity resonance. (c) Extracted near-field profile along *z*-axis (at *x* = 0) for different film thicknesses, clearly highlighting the fast decay of fields in the metal-film.

This near-field also allows the scaling between *I*_bkg_ and *I*_SERS_ to be derived. The decay length (δ) of light confined inside the Au on either side of the gap is obtained from an exponential fit to (*E*/*E*_0_)^2^ for the fields inside the AuNP (Supporting Information, Figure S4). Since the field penetrating the metal then scales as *E*/*t*, the ratio of bgd:SERS ∝ (*Et*^–1^/*E*)^4^·(*t*/*d*) ∝ *t*^–3^, which matches well the experimental data (Figure 3c), with full simulations giving the line shown. Our measurements thus corroborate the MIMI mixing model and indicates that while the field in the gap halves when the foil drops to 10 nm, the field in the dielectric underneath increases 10-fold (at 633 nm, Figure 4). The field null inside the foil is a direct consequence of IMI and MIM mode mixing (Figure 4c). As we derive, the dispersion of coupled MIMI modes shows an in-plane momentum (*k*_∥_), which is larger compared to MIM and IMI modes (Figure 1c), which is what leads to stronger field confinement. This can be intuitively understood from the anticrossing of MIM and IMI modes, which pushes one coupled mode to lower energy (and thus indeed to high wavevectors). Since *k*_⊥_^2^ = *k*_0_^2^ – *k*_∥_^2^, and thus, *k*_⊥_ ≈ *ik*_∥_, modes with a larger wavevector decay faster into the metal, increasing the light confinement. As the foil thickness decreases, the hotspot thus becomes more accessible with improved radiative coupling, but the penetration of light into the metal and the greater field gradient enhances ERS metal scattering. In some experiments, this can cause problems (for instance, S:N values for molecular sensing), while in others, it can prove beneficial, for instance, in nonlinear optical mixing from plasmons.

## Conclusion and Outlook

Having both front and back access to these NPoF systems facilitates improved active control of the material properties assembled into the plasmon proximity. To highlight this, we show here a variety of examples.

**Figure 5:**
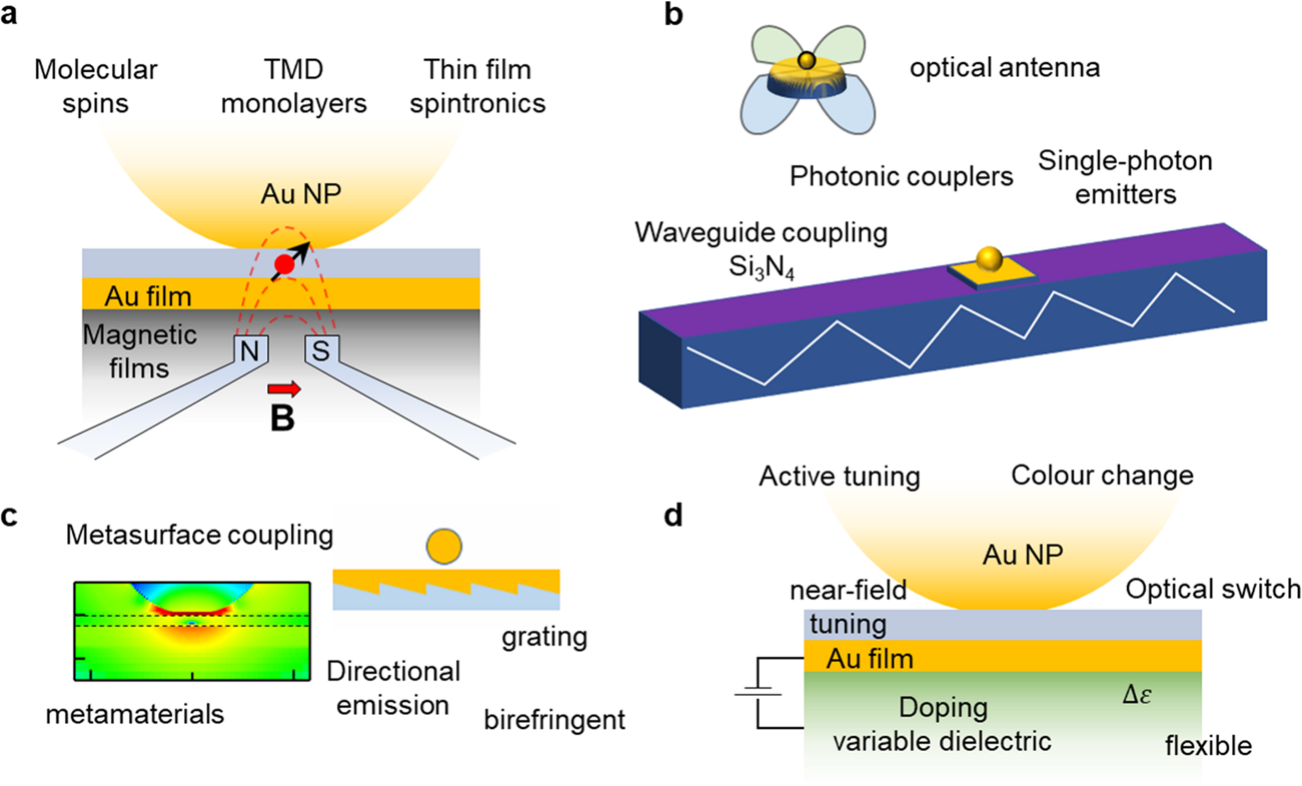
Utilization of NPoF geometry. (a) Coupling of magnetic and plasmonic spin systems. (b) Waveguide integration of NPoFs for single emitters, SERS sensing, and quantum information. (c) Photonic modification of thin metal surface allows coupling of a metasurface to a nanocavity. (d) Doping of underlying dielectric medium changes the MIMI modes and tunes the color as MIMI mixing occurs for *d* ≈ 10 nm gaps.

One long-sought opportunity has been to use magnetic control of the gap material. The NPoF can be assembled onto a magnetic substrate such as iron (Fe) without damping the plasmons when *t* = 10–15 nm (Figure 5a, Supporting Information, Figure S5). This allows a wide variety of magnetoplasmonic applications in controlling molecular and semiconductor spin systems, for example, in transition metal dichalcogenides (TMDs) and other monolayers and bilayers.

Another advantage is providing back access to radiation from NPoFs, allowing their integration into waveguides, which provide coupling to single emitters inside the NPoF gaps (Figure 5b). The combination of the ultranarrow gap geometry providing high Purcell enhancements and the effective coupling to dielectric waveguides makes the system highly viable for quantum optics and sensing.

Importantly, the NPoF construct need not be limited to flat thin Au surfaces. The back-surface of Au can be modified further with photonic/plasmonic metamaterial architectures to fine-tune the IMI modes to create other types of MIMI modes (Figure 5c). This parameter space allows the tuning of the scattered radiation rates and directions and enables new designs incorporating flat lenses. The tunability of this construct makes it compatible to fabricate on flexible surfaces, thus allowing large-scale manufacturing on roll-to-roll systems. As a further opportunity, the dielectric medium underneath the Au surface can also be voltage-tuned (Figure 5d). An example of such an actively changing dielectric would be to integrate 2D materials such as graphene to make this function as a low energy switch.

The nanoparticle-on-foil geometry thus supports new MIMI modes and provides multichannel accessibility to plasmonic hotspots, which gives advantageous opportunities for enhanced integration of extreme plasmonics into nanophotonics devices.

## Methods

### Sample Preparation

Au is deposited directly on a clean SiO_2_ microscope coverslip with a deposition rate of 1 Å/s (LEV Lesker, e-beam evaporator) of different thicknesses after coating with 2 nm of Cr as an adhesive layer. For the realization of single monolayers (SAMs), the Au-coated sample pieces are dipped in a 1 mM solution of biphenyl-4-thiol (BPT, Sigma-Aldrich, 97%) in anhydrous ethanol (Sigma-Aldrich, <0.003% H_2_O) for 12 h. Nanoparticles of 80 nm in diameter (BBI Solutions) are deposited directly onto the BPT-treated Au surface. The deposition time is 15 s.

### Experimental Setup

The sample is placed on a motorized stage (Prior Scientific H101), which is fully automated using an in-house code written in Python. We used an Olympus BX51 microscope with a long working distance ×100 NA 0.8 objective. A spectrally filtered 632.8 nm diode laser (Matchbox, Integrated Optics) with 100 μm/μm^2^ power on the sample and spectral line width of 0.1 pm is used as the excitation pump. In SERS experiments, we filter laser light with a pair of notch filters centered at 633 ± 2 nm (Thorlabs). Inelastically scattered light from the nanoconstructs is coupled through a tube lens into an Andor Shamrock i303 spectrograph and a Newton EMCCD. For dark-field measurements, we used a halogen lamp to excite our samples. Note that we keep the lamp on for around 30 min to stabilize the lamp’s power before starting measurements. The reflected light is collected through the same objective and split to an imaging camera (Lumenera Infinity3–1) and a fiber-coupled spectrometer (Ocean Optics QEPRO) for dark-field spectroscopy.

### Numerical Simulations

Full-wave 3D simulations are performed using Lumerical FDTD Solutions v8.12. The Au NP was modeled as a truncated sphere (with facet width of 20 nm) of a radius of 40 nm on top of an infinite dielectric sheet of refractive index of 1.45 and thickness of 1.3 nm matching the BPT thickness. Underneath the BPT layer are different thicknesses of Au film placed above a thick SiO_2_ substrate. The NPoF is illuminated with plane-waves of polarization perpendicular to the film surface at a glancing 90° angle. The fields scattered into the glass- and air-sides are captured by 2D near-field power monitors.

## Supporting Information Available

(1) Theory of MIM waveguide model and simulation of IMI modes traveling away from the AuNP with different effective indexes. (2) Experimentally measured light scattering from air and glass slide from individual NPoF cavities in modified dual channel microscope. (3) Normal illumination optical characterization of NPoF. (4) Estimating optical field strength inside the metal for MIMI modes. (5) The light scattering from NPoF onto a magnetic substrate (PDF)

## Notes

Source data can be found at DOI link: 10.17863/CAM.72786.
