# Supplementary material for: Accessing Plasmonic Hotspots Using Nanoparticle-on-Foil Constructs
Source: ACS Photonics. 2021 Aug 23;8(9):2811–7. doi: 10.1021/acsphotonics.1c01048 (PMC8447257; doi:10.1021/acsphotonics.1c01048)
Supplement: Supplementary file 1 — ph1c01048_si_001.pdf [file ph1c01048_si_001.pdf]

Supporting Information

Accessing Plasmonic Hotspots using Nanoparticle-on-Foil Constructs

Rohit Chikkaraddy*[1] and Jeremy J Baumberg*[1]
[1] NanoPhotonics Centre, Cavendish Laboratory, Department of Physics, JJ Thompson Avenue, University of Cambridge, Cambridge, CB3 0HE, United Kingdom

A. Theory: MIM waveguide model

The dispersion relation of metal-insulator-metal waveguides with a gap d, is given by the solution of [1]

$$\tanh\left(\frac{\beta_d d}{2}\right) = -\frac{\varepsilon_d \beta_m}{\varepsilon_m \beta_d}$$

where $\beta_{m,d} = \sqrt{k_\parallel^2 - \varepsilon_{m,d} k_0^2}$ for dielectric constants of ε_m (ε_d) in the metal (gap dielectric) and for dielectric constants of ε_m (ε_d) in the metal (gap dielectric) and $k_0 = 2\pi/\lambda$. For the extremely small gaps used here, this expression can be accurately approximated. The MIM waveguide dispersion $E(k_\parallel)$, can be calculated[2,3] for very thin gaps, $d < 10$nm, from

$$\left(\frac{k_\parallel}{E/\hbar c}\right)^2 = \varepsilon_d + \frac{\gamma}{2}\left[1 + \sqrt{1 + 4(\varepsilon_d - \varepsilon_m)/\gamma}\right] \qquad \text{Eqn (S.1)}$$

where $\gamma = \left(\frac{-2\hbar c\, \varepsilon_d}{E\, d\, \varepsilon_m}\right)^2$.

We assume perfect reflection at each end, due to the strong mismatch in impedance within and outside the MIM plasmon waveguide. In practise the phase shift on reflection and the reflectivity of the plasmon depend sensitively on the exact morphology at the facet edges (and this is likely seen in the experiments). However full simulations show that the field strays little outside the facet area for such narrow gaps, and hence this is a reasonable assumption. By using this boundary condition on a circular rim, we find the mode quantisation as given in the main text. We solve simply using Equation (S1) to extract $n_{\rm eff}$ and use this to find the precise wavelengths. An even simpler solution results for the small gap situation, in which $\gamma \gg \varepsilon_m, \varepsilon_d$ so that

$$n_{\rm eff} = -\frac{2\varepsilon_d}{d\varepsilon_m k_0}.$$

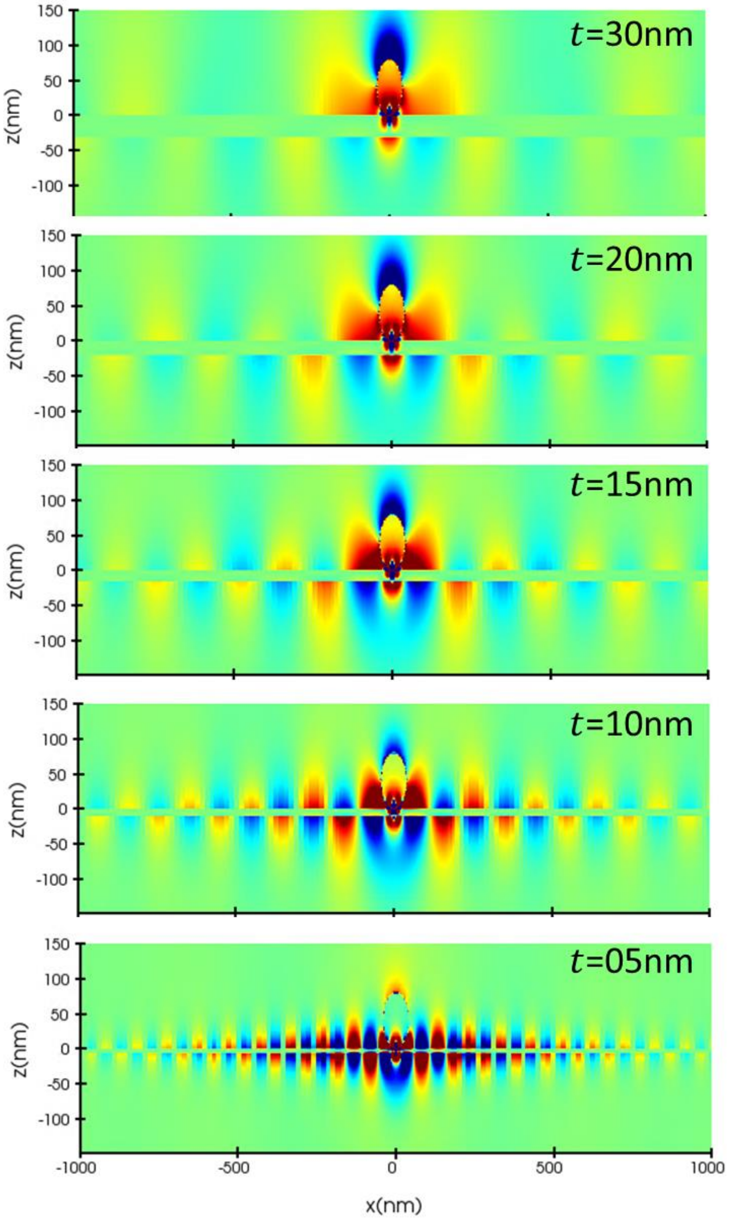

Figure S1. Simulated $\boldsymbol{E_z}$ near-fields for NPoF geometry with vertical dipole source exciting the NPoF gap centre for different foil thickness at λ=633nm, highlighting IMI modes travelling away from the AuNP with different effective index.

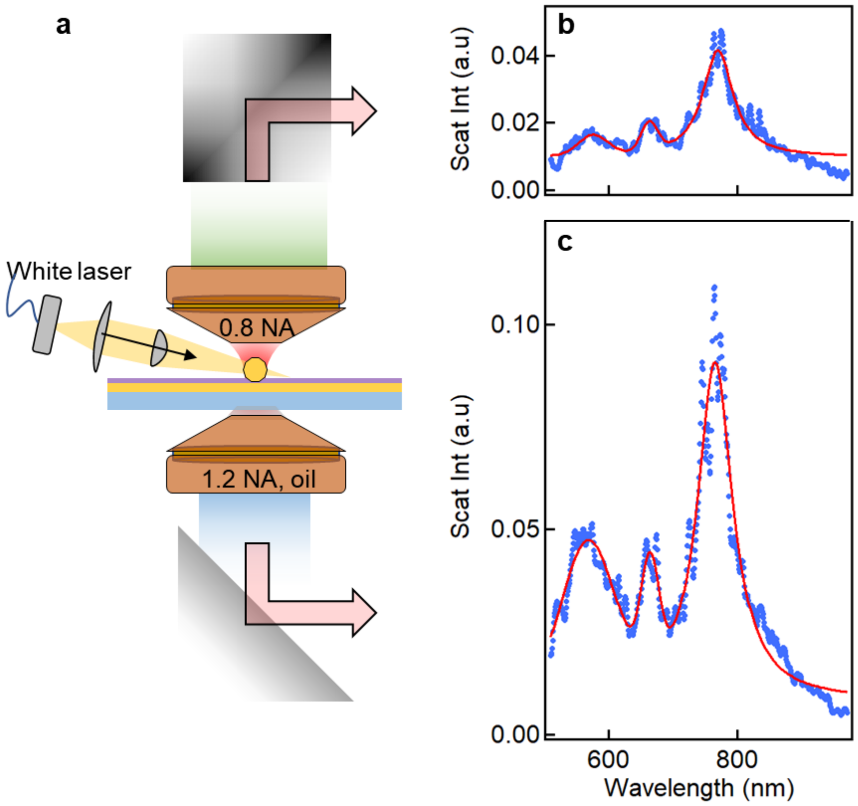

Figure S2. Modified dual channel microscope to collect scattered light from air and glass slide from individual NPoF cavities. (**a**) Schematic of optical microscope with white laser illuminating a wide-area of the sample at high angles and the scattered light collected in two channels (air and glass sides). Experimental scattering spectrum (blue dots) from (**b**) airside and compared to the spectrum obtained from (**c**) the glass side. The data is fit with multiple Lorentzian peaks (red curve) to extract the peak intensity of the lowest order mode.

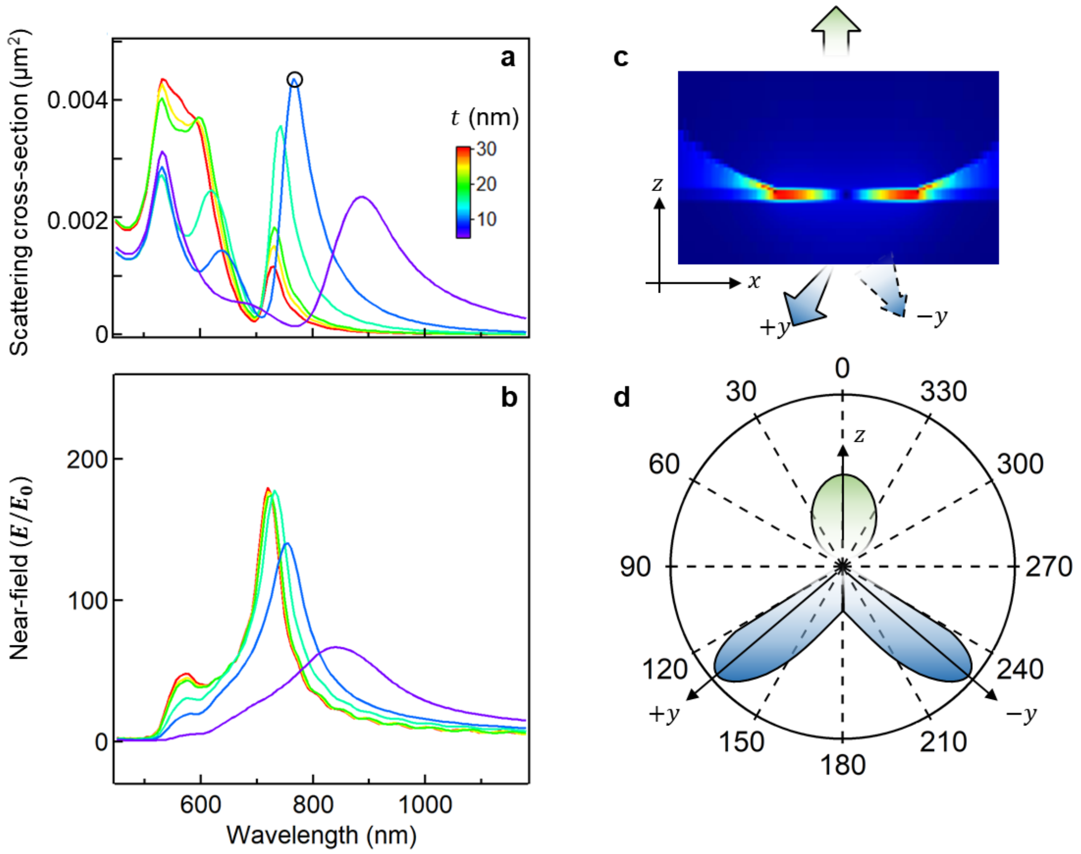

Figure S3. Normal illumination optical characterization of NPoF. (**a**) Scattering spectra for NPoF cavities vs decreasing foil thickness t from 30nm to 5nm. (**b**) Wavelength-dependent near-field intensities extracted from the centre of the BPT-gap layer for different foil thickness. Note that the scattering strength is ×10 weaker in comparison to high-angle illumination (Fig.2c). (**c**) Near-field enhancement map for t=10nm at the nanocavity resonance, indicated by back circle in (a). (**d**) Simulated far-field radiated intensity on a polar plot for NPoF cavity, showing both air-side and glass-side emission. Note that the radiation in the glass side is scattering into the y-direction.

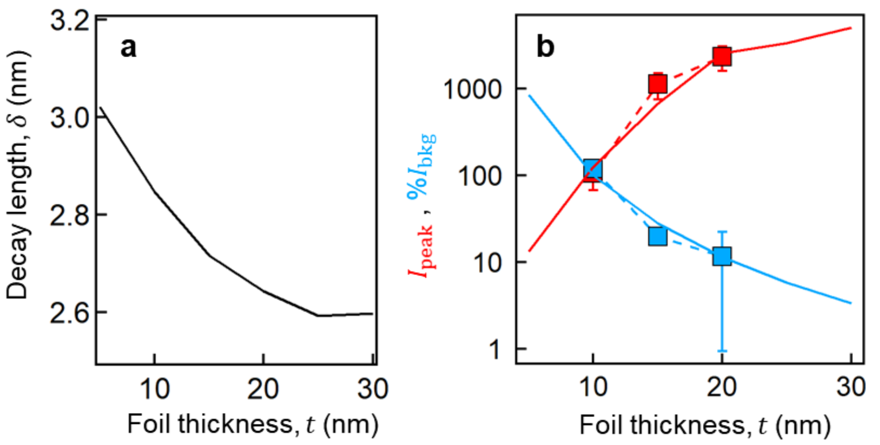

Figure S4. Optical field strength inside the metal for MIMI modes. (**a**) Extracted decay of near-field intensities inside the AuNP as a function of foil thickness. (**b**) Experimentally obtained average SERS peak intensities (I_{peak}, red points) and %SERS background (%I_{bkg}, blue points) obtained from 30 individual NPoF for 3 different film thicknesses. The I_{peak} is fit with $\propto E_{in}^2 . E_{out}^2 . \eta_{air}(t)$, where E_{in}^2 and E_{out}^2 are the electromagnetic field enhancements for the incoming and outcoupling frequencies of light for SERS, respectively. The %I_{bkg} is fit with $\propto \delta/t^3$.

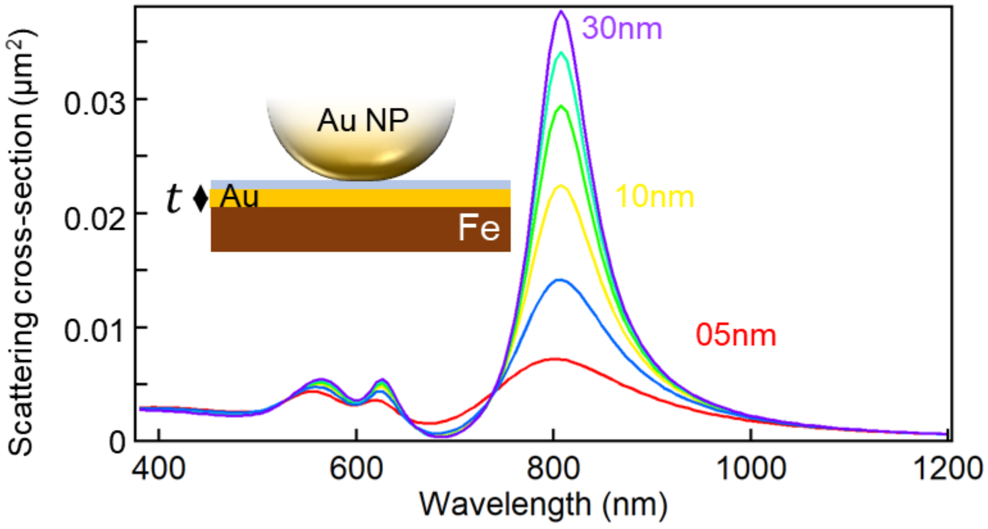

Figure S5. The NPoF can be assembled onto a magnetic substrate such as iron (Fe). The simulated scattering spectra do not exhibit MIMI mode tuning in contrary to SiO_2 substrates for different film thickness and the nanocavity modes are not damped when t>10nm.

References

(1) Sigle, D. O.; Mertens, J.; Herrmann, L. O.; Bowman, R. W.; Ithurria, S.; Dubertret, B.; Shi, Y.; Yang, H. Y.; Tserkezis, C.; Aizpurua, J.; Baumberg, J. J. Monitoring Morphological Changes in 2D

Monolayer Semiconductors Using Atom-Thick Plasmonic Nanocavities. *ACS Nano* **2015**, *9* (1), 825–830. https://doi.org/10.1021/nn5064198.

(2) Bozhevolnyi, S. I.; Søndergaard, T. General Properties of Slow-Plasmon Resonant Nanostructures: Nano-Antennas and Resonators. *Opt. Express* **2007**, *15* (17), 10869–10877. https://doi.org/10.1364/OE.15.010869.

(3) Kuttge, M.; Cai, W.; García de Abajo, F. J.; Polman, A. Dispersion of Metal-Insulator-Metal Plasmon Polaritons Probed by Cathodoluminescence Imaging Spectroscopy. *Phys. Rev. B* **2009**, *80* (3), 033409. https://doi.org/10.1103/PhysRevB.80.033409.
